# Supplementary material for: Modeling glioblastoma heterogeneity as a dynamic network of cell states
Source: Mol Syst Biol. 2021 Sep 16;17(9):e10105. doi: 10.15252/msb.202010105 (PMC8444284; doi:10.15252/msb.202010105)
Supplement: Supplementary file 5 — Source Data for Figure 3 [file MSB-17-e10105-s001.zip › Figure3A_sourcedata/GSEA_3065/hallmarks_state3.GseaPreranked.1623416274744/HALLMARK_UV_RESPONSE_DN.html]

Details for gene set HALLMARK\_UV\_RESPONSE\_DN[GSEA]

|  || Dataset | state3 |
| Phenotype | NoPhenotypeAvailable |
| Upregulated in class | na\_pos |
| GeneSet | HALLMARK\_UV\_RESPONSE\_DN |
| Enrichment Score (ES) | 0.35870966 |
| Normalized Enrichment Score (NES) | 1.4166819 |
| Nominal p-value | 0.009375 |
| FDR q-value | 0.08990933 |
| FWER p-Value | 0.391 |
Table: GSEA Results Summary

  

Fig 1: Enrichment plot: HALLMARK\_UV\_RESPONSE\_DN      
 Profile of the Running ES Score & Positions of GeneSet Members on the Rank Ordered List

  

| PROBE | GENE SYMBOL | GENE\_TITLE | RANK IN GENE LIST | RANK METRIC SCORE | RUNNING ES | CORE ENRICHMENT || 1 | MMP16 |  |  | 94 | 0.351 | 0.0289 | Yes |
| 2 | LAMC1 |  |  | 96 | 0.349 | 0.0684 | Yes |
| 3 | TJP1 |  |  | 139 | 0.314 | 0.0991 | Yes |
| 4 | COL5A2 |  |  | 266 | 0.255 | 0.1134 | Yes |
| 5 | ATP2B1 |  |  | 271 | 0.253 | 0.1416 | Yes |
| 6 | PRKCA |  |  | 328 | 0.234 | 0.1617 | Yes |
| 7 | TFPI |  |  | 480 | 0.202 | 0.1671 | Yes |
| 8 | LDLR |  |  | 498 | 0.199 | 0.1876 | Yes |
| 9 | PMP22 |  |  | 556 | 0.192 | 0.2028 | Yes |
| 10 | HAS2 |  |  | 603 | 0.186 | 0.2185 | Yes |
| 11 | ATRX |  |  | 638 | 0.180 | 0.2350 | Yes |
| 12 | ATP2C1 |  |  | 671 | 0.175 | 0.2512 | Yes |
| 13 | CDK13 |  |  | 681 | 0.174 | 0.2698 | Yes |
| 14 | COL11A1 |  |  | 684 | 0.173 | 0.2892 | Yes |
| 15 | PLPP3 |  |  | 786 | 0.159 | 0.2956 | Yes |
| 16 | ATRN |  |  | 800 | 0.157 | 0.3119 | Yes |
| 17 | MAP1B |  |  | 975 | 0.140 | 0.3077 | Yes |
| 18 | CDC42BPA |  |  | 1141 | 0.127 | 0.3030 | Yes |
| 19 | GJA1 |  |  | 1160 | 0.125 | 0.3151 | Yes |
| 20 | RUNX1 |  |  | 1221 | 0.120 | 0.3217 | Yes |
| 21 | SIPA1L1 |  |  | 1377 | 0.110 | 0.3163 | Yes |
| 22 | ARHGEF9 |  |  | 1402 | 0.108 | 0.3257 | Yes |
| 23 | NIPBL |  |  | 1434 | 0.106 | 0.3342 | Yes |
| 24 | TENT4A |  |  | 1696 | 0.092 | 0.3144 | Yes |
| 25 | SYNE1 |  |  | 1729 | 0.090 | 0.3210 | Yes |
| 26 | FZD2 |  |  | 1774 | 0.088 | 0.3258 | Yes |
| 27 | SLC22A18 |  |  | 1782 | 0.087 | 0.3349 | Yes |
| 28 | SDC2 |  |  | 1875 | 0.082 | 0.3336 | Yes |
| 29 | SLC7A1 |  |  | 1936 | 0.079 | 0.3356 | Yes |
| 30 | ERBB2 |  |  | 1987 | 0.077 | 0.3385 | Yes |
| 31 | NOTCH2 |  |  | 1990 | 0.076 | 0.3469 | Yes |
| 32 | APBB2 |  |  | 2011 | 0.076 | 0.3532 | Yes |
| 33 | IGF1R |  |  | 2037 | 0.074 | 0.3587 | Yes |
| 34 | SFMBT1 |  |  | 2211 | 0.068 | 0.3464 | No |
| 35 | EFEMP1 |  |  | 2265 | 0.066 | 0.3477 | No |
| 36 | MYC |  |  | 2324 | 0.064 | 0.3482 | No |
| 37 | DYRK1A |  |  | 2413 | 0.060 | 0.3448 | No |
| 38 | KALRN |  |  | 2432 | 0.059 | 0.3495 | No |
| 39 | ATP2B4 |  |  | 2510 | 0.057 | 0.3470 | No |
| 40 | MET |  |  | 2522 | 0.056 | 0.3521 | No |
| 41 | DLG1 |  |  | 2526 | 0.056 | 0.3581 | No |
| 42 | VLDLR |  |  | 2734 | 0.049 | 0.3397 | No |
| 43 | BMPR1A |  |  | 2736 | 0.049 | 0.3452 | No |
| 44 | MRPS31 |  |  | 2774 | 0.048 | 0.3463 | No |
| 45 | ADD3 |  |  | 2929 | 0.043 | 0.3334 | No |
| 46 | PRDM2 |  |  | 3027 | 0.040 | 0.3268 | No |
| 47 | MIOS |  |  | 3032 | 0.040 | 0.3309 | No |
| 48 | VAV2 |  |  | 3078 | 0.039 | 0.3301 | No |
| 49 | SERPINE1 |  |  | 3090 | 0.039 | 0.3332 | No |
| 50 | INSIG1 |  |  | 3136 | 0.038 | 0.3322 | No |
| 51 | CAP2 |  |  | 3313 | 0.033 | 0.3156 | No |
| 52 | MAGI2 |  |  | 3399 | 0.031 | 0.3092 | No |
| 53 | PTEN |  |  | 3464 | 0.029 | 0.3051 | No |
| 54 | PHF3 |  |  | 3468 | 0.029 | 0.3080 | No |
| 55 | ATXN1 |  |  | 3504 | 0.028 | 0.3072 | No |
| 56 | IGFBP5 |  |  | 3555 | 0.027 | 0.3044 | No |
| 57 | ACVR2A |  |  | 3557 | 0.027 | 0.3073 | No |
| 58 | SMAD3 |  |  | 3767 | 0.021 | 0.2855 | No |
| 59 | RXRA |  |  | 3769 | 0.021 | 0.2877 | No |
| 60 | TGFBR2 |  |  | 3803 | 0.020 | 0.2862 | No |
| 61 | PIK3R3 |  |  | 3885 | 0.018 | 0.2789 | No |
| 62 | SCHIP1 |  |  | 4109 | 0.012 | 0.2544 | No |
| 63 | DUSP1 |  |  | 4186 | 0.010 | 0.2468 | No |
| 64 | SCAF8 |  |  | 4288 | 0.007 | 0.2360 | No |
| 65 | PDLIM5 |  |  | 4368 | 0.005 | 0.2274 | No |
| 66 | DMAC2L |  |  | 4403 | 0.004 | 0.2239 | No |
| 67 | IRS1 |  |  | 4495 | 0.001 | 0.2135 | No |
| 68 | RASA2 |  |  | 4517 | 0.001 | 0.2112 | No |
| 69 | NFKB1 |  |  | 4528 | 0.001 | 0.2101 | No |
| 70 | CACNA1A |  |  | 4871 | -0.008 | 0.1714 | No |
| 71 | ICA1 |  |  | 5053 | -0.012 | 0.1519 | No |
| 72 | AMPH |  |  | 5081 | -0.013 | 0.1502 | No |
| 73 | YTHDC1 |  |  | 5158 | -0.015 | 0.1431 | No |
| 74 | ADORA2B |  |  | 5176 | -0.015 | 0.1428 | No |
| 75 | BCKDHB |  |  | 5245 | -0.017 | 0.1368 | No |
| 76 | BHLHE40 |  |  | 5331 | -0.019 | 0.1292 | No |
| 77 | MAPK14 |  |  | 5663 | -0.029 | 0.0942 | No |
| 78 | NR3C1 |  |  | 5724 | -0.030 | 0.0907 | No |
| 79 | NR1D2 |  |  | 5753 | -0.031 | 0.0910 | No |
| 80 | CDKN1B |  |  | 5783 | -0.032 | 0.0913 | No |
| 81 | MAP2K5 |  |  | 5811 | -0.033 | 0.0920 | No |
| 82 | SYNJ2 |  |  | 5836 | -0.034 | 0.0930 | No |
| 83 | BDNF |  |  | 5896 | -0.036 | 0.0902 | No |
| 84 | AKT3 |  |  | 5940 | -0.037 | 0.0895 | No |
| 85 | DDAH1 |  |  | 6076 | -0.042 | 0.0786 | No |
| 86 | AGGF1 |  |  | 6080 | -0.042 | 0.0830 | No |
| 87 | CELF2 |  |  | 6152 | -0.045 | 0.0799 | No |
| 88 | SPOP |  |  | 6228 | -0.047 | 0.0766 | No |
| 89 | ZMIZ1 |  |  | 6296 | -0.050 | 0.0745 | No |
| 90 | RBPMS |  |  | 6497 | -0.056 | 0.0577 | No |
| 91 | NFIB |  |  | 6540 | -0.058 | 0.0594 | No |
| 92 | MTA1 |  |  | 6668 | -0.064 | 0.0520 | No |
| 93 | CAV1 |  |  | 6859 | -0.073 | 0.0384 | No |
| 94 | FYN |  |  | 6924 | -0.077 | 0.0397 | No |
| 95 | PRKAR2B |  |  | 7104 | -0.088 | 0.0289 | No |
| 96 | FHL2 |  |  | 7163 | -0.091 | 0.0325 | No |
| 97 | RND3 |  |  | 7179 | -0.092 | 0.0413 | No |
| 98 | CITED2 |  |  | 7430 | -0.111 | 0.0249 | No |
| 99 | KCNMA1 |  |  | 7520 | -0.119 | 0.0281 | No |
| 100 | MGLL |  |  | 7773 | -0.148 | 0.0157 | No |
| 101 | ANXA4 |  |  | 7847 | -0.158 | 0.0252 | No |
| 102 | ID1 |  |  | 7938 | -0.175 | 0.0346 | No |
| 103 | ANXA2 |  |  | 8206 | -0.230 | 0.0298 | No |
| 104 | SRI |  |  | 8395 | -0.292 | 0.0412 | No |
Table: GSEA details [plain text format]

  

Fig 2: HALLMARK\_UV\_RESPONSE\_DN: Random ES distribution      
 Gene set null distribution of ES for **HALLMARK\_UV\_RESPONSE\_DN**
